# On the Chemistry of Digestion

**Published:** 1853

**Authors:** G. E. Day


### *On the Chemistry of Digestion.*—By Dr. G. E. Day.

[Our readers can hardly fail to appreciate the following elaborate review of Professors Bidder and Schmidt's work on 'The Digestive Fluids and Metamorphosis of Tissue,' and of Dr. Carpenter's 'Food and Digestive Process,' by Dr. G. E. Day, the esteemed Professor of medicine at St. Andrews. Of the different secretions employed in the digestive process, that of the salivary glands comes naturally to be considered first. The principal use of this would appear to be to promote the conversion of the amylacea into dextrine, sugar, and lactic acid, and thus promote the absorption of this class of foods.]

The first point we shall notice is the time required for metamorphosis of starch by human saliva (the mixed secretion). If we take a fresh decoction of starch, prepared with distilled water, and proved by Trommer's test to be free from sugar, and if we mix it with an equal quantity of fresh saliva, and agitate the mixture, it will instantly lose its viscid character, and become thin and watery; on testing a small quantity of it for starch, we find that iodine no longer induces the well-known reaction, while, on the other hand, the rapid reduction of oxide of copper (in Trommer's test) affords indisputable evidence of the presence of sugar.

The almost instantaneous induction of this action is a point which must not be overlooked, in considering the question whether this is a special property of the saliva, or whether it is shared by other animal fluids. There can be no doubt, as we shall presently show, that in this respect the pancreatic and the intestinal juices exactly coincide with the saliva; but when we find stress laid upon the circumstance that many organic substances—as, for instance, nasal mucus, pieces of kidney, putrefying serum, &c.—produce similar changes in eight or twelve hours, at 100 degrees or upwards, we may recollect that at such a temperature, and after so long an interval, changes may be spontaneously set up in a solution of starch. There are, however, a number of animal substances which occasion the appearance of sugar in a solution of starch, in so short a period as altogether to exclude, in such cases, the suspicion of spontaneous metamorphosis; but the action induced by the saliva is incomparably more rapid even than that of any of these substances.

[We may ask, are the secretions of the three sets of salivary glands of equal importance in producing the final result?]

The following are the two principal conclusions at which Bidder and Schmidt have arrived in connection with these points:

1. They agree with Bernard in regarding the parotids as *glandes equipares;* in short, as yielding a secretion which is unquestionably intended to moisten and saturate the dry food, but whose principal object is connected with the general metamorphosis of the fluids within the body, and which is devoid of any marked action on starch.

2. By the union of the submaxillary secretion and that of the buccal mucous membrane, there is formed that peculiar ferment which almost instantaneously converts starch into sugar. This active principle is not contained in the cells or other solid particles suspended in the saliva; for the filtered fluid exhibits an undiminished force; and, indeed, this property is not destroyed when, by the addition of a little alcohol, we precipitate the mucus and (entangled in it) these solid particles.

There is still considerable obscurity and diversity of opinion regarding the question, whether the action of the saliva on starch is continued in the stomach. Jacubowitsch, Frerichs, and Bence Jones, believe the metamorphic process is continued in the stomach, and Lehman and Carpenter support this view, while the experiments of Bidder and Schmidt lead to an opposite result.

[It would appear from these experiments that starch should be rapidly converted into sugar in the stomach; but in animals, where it has been introduced artificially, the result has afforded a conclusive demonstration that no converson into sugar takes place in this organ.]

Now, seeing, in the first place, that the formation of sugar commences in the mouth immediately upon the starch coming in contact with the saliva, and further bearing in mind that no sugar can be usually detected in the stomach, it follows that one of two things must occur—either that the newly-formed sugar is at once absorbed, or that it is converted into lactic acid, and in this form mixes with and is lost in the gastric juice. In the latter case, we must ascribe to the gastric mucous membrane the power of instantaneously converting sugar into lactic acid—a power which, as we learn by experiments, is possessed neither by the stomachs of herbivora or carnivora; in the former case, we can only say, that so rapid an absorption is perhaps the least improbable mode of explanation.

We are bound to remark, that the above opinion of Bidder and Schmidt, regarding the absence of sugar in the gastric contents of animals fed upon starch, has not met with universal acceptation. It is true that it is in accordance with views previously expressed by Blondlot, and by Bouchardat and Sandras, but is opposed to the results obtained by Frerichs (who "in at least fifty experiments" constantly found sugar in the filtered contents of the stomachs of men, carnivorous and omnivorous mammalia, and birds), and to those of Jacubowitsch.

The secretion of the glandular apparatus imbedded in and forming the chief bulk of the mucous coat of the stomach next claims our attention. Many of our best recent observers (amongst whom we may especially mention Hubbenet, a pupil of Bidder and Schmidt's) believe that two perfectly distinct secretions are found in the stomach—namely, the true gastric juice and gastric mucus. Almost all observers, since Beaumont, have agreed in describing the former as a clear aqueous or only slightly viscid fluid, with a strong acid reaction, and devoid of morphological elements—that is to say, of essential morphological elements; for on a microscopic examination, a few solid particles, consisting partly of unchanged cells of the gastric glands, partly of the nuclei of these cells, and partly of fine molecular matter, which is produced by the disintegration of these elements, may usually be observed; it is only secreted during the period when digestion is actually going on, and it is this fluid alone which possesses any true digestive power. All that is known of the latter (the mucus) is, that it accumulates in the empty stomach, that it is neutral (unless when slightly alkaline from the commixture of saliva), and that it exerts no solvent action on the protein compounds. Frerichs, however, whose views on the chemistry of digestion are entitled to the highest respect, holds a different view, and maintains that at the commencement of each act of digestion the round cells in the interior of the glands escape in excessive quantities, and form a stratum of about a line in thickness, which has been hitherto regarded as mucus, and which either invests the interior of the stomach, or surrounds the contents in the form of a white membrane, the latter being especially the case when dry food is taken. The gastric cells become gradually disintegrated during the continuance of the digestive process, and thus afford a continuous source of pepsin or ferment. The digestive act being accomplished, the gastric glands collapse, and in that state no nuclei or cells, and only a few scattered granules, escape from them. During abstinence from food, the morphological elements are again perfectly formed, and the tubes become filled with cells, which probably in very prolonged fasting again become disintegrated.

There two points in connexion with the secretions of the gastric mucous membrane to which Kolliker has directed special attention, and which we will now lay in a condensed form before our readers.

The first is, that in many of the mammalia the gastric mucous membrane is covered with a more or less thick coat of mucus during digestion. This fact was originally noticed by Eberle in the stomachs of rabbits, and he clearly regarded the mucus as essential to digestion.

[The gastric mucus can scarcely be regarded as essential to the digestive process, except that it may possibly exert a peculiar influence in certain parts of the stomach. The gastric juice is acid, unless there be an excess of mucus, when it may be neutral, or when a large quantity of saliva is swallowed, when it may be alkaline. It contains a free acid, a highly nitrogenized matter, termed 'pepsin,' or, more recently styled, 'ferment substance.' It is perfectly astonishing how enormous have been the labours of these indefatigable labourers, MM. Bidder and Schmidt. They have recorded no less than 45 series of experimental inquiries, embracing 140 individual experiments.]

Their experiments lead to the following conclusions:—

The admixture of saliva with the gastric juice is of no service, in so far as the solution of albuminous bodies is concerned. It appears, that in experiments with gastric juice containing saliva, the number expressing the dissolved per centage of dry albumen was not so high as in corresponding experiments (both sets of experiments being made within the stomachs of dogs) when the saliva was excluded. Whilst in the latter the averages of nine experiments of two hours, nine of four hours, and nine of six hours duration were 29, 62, and 76 per cent., in the former they were only 26, 45, and 65 per cent. This difference is unquestionably dependent upon the partial neutralization of the free acid of the gastric juice by the saliva.

Both classes of experiments agree in showing that the acid gastric juice possesses a specific and peculiar power of dissolving coagulated albumen; and if this solution proceeds with more rapidity in the stomach than externally to the organism, the reason is, that in the former the albumen is kept in constant motion, and is being always brought into contact with fresh quantities of gastric juice. Several of Bidder and Schmidt's experiments show, that provided a sufficient quantity of pepsin be present, the digestive powei of the gastric juice varies in a direct ratio with the amount of free acid that it contains. That the solvent power of the gastric juice is not solely due to the presence of hydro-chloric acid is, however, evident from the results of several experiments, which showed that this acid, in a diluted state, could only, at the most, exert one-fourth part of the solvent action on coagulated albumen which was exerted by the gastric juice itself. The neutral or alkaline gastric mucus of fasting animals dissolved only very slight quantities of albumen, but on adding hydrochloric acid the activity of the fluid was at once augmented. On neutralizing the free acid of the gastric juice with potash, its digestive power is destroyed.

With regard to the effects of filtration, they observe that repeated experiments have convinced them that gastric juice from which all undissolved organic matters have been separated acts with the same efficiency as that which has not been filtered. This is obviously at variance with the view held by Frerichs, that new ferment continues to be developed from the cells contained in the gastric juice by the action of the free acid.

The experiments on concentrated and diluted gastric juice led to no definite result: indeed, no experiments seem to have been made with the concentrated fluid till it had been re-diluted with water.

It was proved by several experiments that the solvent action of the gastric juice is destroyed by rapid ebullition, or by evaporation at a boiling heat—an additional illustration of the well-known fact, that ferments are decomposed at an elevated temperature. This may possibly be the basis of the universally recognized dietetic rule, which declares all very hot foods or drinks to be injurious. On the the other hand, the solidification of the gastric juice by artificial cold did not destroy its solvent powers—a circumstance which may be consolatory to those who are addicted to ices.

It was distinctly shown by three experiments, that the admixture of bile with the gastric juice—even when the quantity is so small that the mixture retains a distinctly acid reaction—completely destroys the solvent power of the latter secretion upon coagulated albumen.

The quantity of the gastric juice secreted in twenty-four hours was determined by Bidder and Schmidt, in the case of dogs, to be about one-tenth of the whole weight of the body. Assuming the same ratio to be true for men, a man of ordinary weight (say from ten to twelve stone) would daily secrete no less than from fourteen to about seventeen pounds of this fluid. This is a very different estimate from that formed by Lehmann, whose calculations were based on the following data:—

100 grammes of the fresh gastric juice of a dog connot, according to Lehman's experiments, effect the solution of more than 5 grammes of coagulated albumen (calculated as dry).

An adult man receives into the stomach about 100 grammes of dry albuminous matter in twenty-four hours.

Hence, to digest this quantity, there must be secreted 2000 grammes, or four pounds of gastric juice.

We now proceed to the consideration of the bile—a subject to which Bidder and Schmidt have devoted much experimental labor.

The first question they consider is the following:—Is the bile to be regarded simply as an excretion? This point they have attempted to decide by observations on dogs in whom billiary fistulæ had been established.

A dog on whom this operation was performed lost two-fifths of its weight in thirty-four days, although its appetite was increased rather than diminished. The emaciation was especially marked both in the loss of fat and muscle. The hair fell off at many spots, or was loosened by the slightest touch. Digestion appeared to go on in an undisturbed manner; although the animal was in the habit of licking the bile as it escaped. The excrements had a greyish-yellow, and sometimes a partially green color; but during the last few days of life regained their normal color and odor. There were constant eructations of a bad odor, and the breath likewise had a very unpleasant smell. The urine was occasionally so concentrated, that its specific gravity was 1.050. The animal at length (on the thirty-fourth day) died from general exhaustion. Dissection showed that a minute new ductus choledochus had become formed, probably between the twenty-first and twenty-fifth day after the operation, at which time the fistulous opening presented a tendency to contract. The right half of the stomach presented numerous ecchymoses, both of older and of more recent standing, although there were no symptoms of disturbed gastric digestion during life—that is to say, there was no vomiting, and the appetite continued unimpaired. The rest of the intestinal tract presented nothing abnormal, except that it was of a somewhat deeper red tint than usual.

A second dog, similarly treated, did not present a voracious appetite, or exhibit the same amount of flatulence, or such a tendency to putrefactive decomposition as the former animal; but in the course of twenty-seven days (when it died) it had lost about one-half of its weight. Except that the fat and the muscles had almost disappeared, none of the organs were found to present any marked peculiarity.

Since the quantity of bile that escaped from the system in these two cases was not compensated for by the additional food that was taken, the idea suggested itself that the emaciation and the disturbed condition of the metamorphosis going on within the tissues might be due to the continuous drain upon the system. This view was confirmed by the two following experiments, in which this disturbing cause was eliminated.

A dog, whose spleen had been extirpated, and in whom a biliary fistula had been established fourteen days afterwards, took such large quantities of food as more than to compensate for what was lost by the escape of bile. Its bodily weight, under these circumstances, did not materially diminish during the space of two months, at the end of which time it was killed. The normal development and power of the muscles were retained, but the fat had gradually disappeared. Putrefactive decomposition of the excrements, and abundant flatus of a disgusting odor, were likewise observed in this case.

A very emaciated dog, whose spleen had not been extirpated, and in whom a biliary fistula had been established, regained its healthy appearance and its previous normal weight nineteen days after the operation. During this period it took more meat and bread than were requisite for it in its perfect healthy condition. The ductus choledochus subsequently became re-established, and the weight then again materially increased.

From these experiments it seems clear that the question which they attempt to solve cannot be directly and positively answered either in the affirmative or the negative. By an artificial interference with the organism (by the establishment of a biliary fistula) the bile may be reduced, in a certain sense, to the condition of an excretion, if the loss thus occasioned to the system at large can be suitably covered. If this compensation be impossible, the derivation of the bile from the intestinal canal, and its direct external discharge in a comparatively short time induce fatal emaciation and debility. For the full comprehension of the question in all its bearings, we should know the quantity of bile secreted in a given time, and the average amount of solid constituents which it contains, the share which it takes in the process of digestion, the changes which it undergoes during and after its resorption into the blood, and lastly, the form in which it is finally eliminated from the organism.

In the strict sense of the word, we can hardly regard the bile as a digestive fluid. There is no special class of substances whose solution is dependent upon it. If, as certain experiments show, animals with biliary fistula can live even for years, the function of this fluid in digestion must, at all events, be a limited, and probably only an indirect one; and this is confirmed by the fact, that the secretion of bile does not attain its maximum till twelve or fifteen hours after food has been taken, when by far the greatest part of the ingesta must have passed beyond the duodenum; hence the greatest part of this fluid enters the small intestine at much too late a period to exert in it any great influence on the metamorphosis of the chyme.

[From trials upon various animals it has been ascertained that although the pancreatic juice exerts no solvent action in albuminous bodies, it yet acts with extreme power in converting starch into dextrine and sugar.]

Bernard, about four years ago, discovered that remarkable fact, that, externally to the organism, the pancreatic juice possesses the property of decomposing the neutral fats into their base and acid.

The only unquestionable action of the pancreatic fluid is that which it exerts on starch; but since the relative size of the pancreas is greater in carnivorous than in herbivorous animals (the weight of this gland being one-three hundredth that of the whole body in dogs and cats, and only one-six-hundredth in that of rabbits), and since, further [as has been shown by Bidder and Schmidt's experiments], the greater part of the amylaceous food of the sheep is converted into sugar before it enters the duodenum, we may fairly conclude that the principal uses of this secretion are still unknown.

There are far greater difficulties in obtaining pure intestinal juice than in procuring any of the secretions we have previously considered. The most recent investigations on this subject are those of Frerichs [contained in his article on digestion], and of Zander [a pupil of Bidder and Schmidt's, who have themselves continued and extended his researches], and their conclusions are, upon the whole, very discordant.

All that we can say with certainty with regard to the physico-chemical properties of the fluid is, that, after filtration, it is a tenacious, ropy, colorless, and strongly alkaline substance.

According to Frerichs, the intestinal juice exerts no change on any of the ordinary elements of food. Protein bodies and gelatigenous substances remained perfectly unchanged; fat became disintegrated in the same manner as in all other viscid fluids; neither, according to Frerichs, does it exert any special action on starch. Hence he denies to the intestinal fluid any action as a direct digestive agent. Lehmann, on the other hand, found that the intestinal juice possessed, in a high degree, the power of converting starch into sugar; but protein bodies and fats were so little affected by it, that he doubts whether it exerts any digestive action on these substances, and the more so, since cubes of coagulated albumen and pieces of flesh, when introduced into the gut of a hospital patient through a fistulous opening, were expelled from the rectum almost entirely unchanged. Tho fistula was, however, in the lower part of the ileum, and probably near the cæcum. Finally, the results of experiments on nineteen cats and two dogs have convinced Zander and Bidder and Schmidt, that the intestinal juice exerts a solvent action on albuminous bodies, scarcely inferior to that of the gastric juice, and a sugar-forming power on starch scarcely inferior to that of saliva or of pancreatic juice.

*Brit. and For. Med. Chir. Review*, July, 1853, p. 171.

